# Methotrexate-loaded nanoparticles ameliorate experimental model of autoimmune arthritis by regulating the balance of interleukin-17-producing T cells and regulatory T cells

**DOI:** 10.1186/s12967-022-03267-0

**Published:** 2022-02-11

**Authors:** Jin-Sil Park, Donghyun Lee, SeungCheon Yang, Ha Yeon Jeong, Hyun Sik Na, Keun-Hyung Cho, JeongWon Choi, Heebeom Koo, Mi-La Cho, Sung-Hwan Park

**Affiliations:** 1 The Rheumatism Research Center, Catholic Research Institute of Medical Science, College of Medicine, The Catholic University of Korea, 222, Banpo-daero, Seocho-gu, Seoul, 06591 Republic of Korea; 2 Department of Medical Life Sciences, College of Medicine, The Catholic University of Korea, 222 Banpo-daero, Seocho-gu, Seoul, 06591 Republic of Korea; 3 Department of Biomedicine and Health Sciences, College of Medicine, The Catholic University of Korea, 222 Banpo-daero, Seocho-gu, Seoul, 06591 Republic of Korea; 4 Division of Rheumatology, Department of Internal Medicine, School of Medicine, The Catholic University of Korea, Seoul St. Mary’s Hospital, 222 Banpo-Daero, Seocho-gu, Seoul, 137-701 South Korea; 5 Lab of Translational ImmunoMedicine, Catholic Research Institute of Medical Science, College of Medicine, College of Medicine, The Catholic University of Korea, Seoul, Republic of Korea

**Keywords:** Rheumatoid arthritis, Nanoparticles, Methotrexate, Interleukin-17-producing T cells, Regulatory B cells

## Abstract

**Background:**

Rheumatoid arthritis (RA) is a progressive systemic autoimmune disease that is characterized by infiltration of inflammatory cells into the hyperplastic synovial tissue, resulting in subsequent destruction of adjacent articular cartilage and bone. Methotrexate (MTX), the first conventional disease-modifying antirheumatic drug (DMARD), could alleviate articular damage in RA and is implicated in humoral and cellular immune responses. However, MTX has several side effects, so efficient delivery of low-dose MTX is important.

**Methods:**

To investigate the efficacy of MTX-loaded nanoparticles (MTX-NPs) against experimental model of RA, free MTX or MTX-NPs were administered as subcutaneous route to mice with collagen-induced arthritis (CIA) at 3 weeks after CII immunization. The levels of inflammatory factors in tissues were determined by immunohistochemistry, confocal microscopy, real-time PCR, and flow cytometry.

**Results:**

MTX-NPs ameliorated arthritic severity and joint destruction in collagen-induced arthritis (CIA) mice compared to free MTX-treated CIA mice. The levels of inflammatory cytokines, including interleukin (IL)-1β, tumor necrosis factor-α, and vascular endothelial growth factor, were reduced in MTX-NPs-treated mice. Number of CD4 + IL-17 + cells decreased whereas the number of CD4 + CD25 + Foxp3 + cells increased in spleens from MTX- NPs-treated CIA mice compared to MTX-treated CIA mice. The frequency of CD19 + CD25 + Foxp3 + regulatory B cells increased in ex vivo splenocytes from MTX-loaded NPs-treated CIA mice compared to MTX-treated CIA mice.

**Conclusion:**

The results suggest that MTX-loaded NPs have therapeutic potential for RA.

## Background

Rheumatoid arthritis (RA) is a progressive autoimmune disease characterized by synovial inflammation, hyperplasia, and formation of rheumatoid pannus, resulting in destruction of the adjacent articular cartilage and bone structure. The development of RA involves a complex interplay between immune cells and inflammatory mediators including inflammatory cytokines, proteolytic enzymes, and prostanoids [1–3]. Although the causes of RA are unclear, infiltration of T and B cells into the joints leads to induction of inflammatory cytokines, such as tumor necrosis factor (TNF)-α, interleukin (IL)-6, IL-1β, and IL-17A and autoantibodies and drives the proliferation of fibroblast-like synoviocytes and destruction of bone [3, 4].

IL-17-producing T helper (Th17) cells, which produce IL-17A, IL-17F, IL-21, and TNF-α, are involved in the pathogenesis of various autoimmune diseases, including RA, multiple sclerosis, and psoriasis [5, 6]. IL-6 drives the differentiation of naïve T cells into Th17 cells by activating STAT3, a key transcription factor for Th17 cells [7, 8]. In RA synovium, IL-17-producing cells were observed in the T cell-rich area and IL-17 contributes to increased production of IL-6 and leukemia inhibitory factor. Moreover, it has synergistic effects with IL-1β or TNF-α on inflammation [9–11]. Rheumatoid synovial tissue contains abundant B cells, which secrete proinflammatory mediators and autoantibodies, including rheumatoid factor and anti–citrullinated protein antibodies [3, 12]. Moreover, B cells act as antigen-presenting cells for autoreactive T cells [13].

Methotrexate (MTX) is a first-line disease-modifying antirheumatic drug (DMARD) that alleviates articular damage in RA [14]. It is an antifolate metabolite that inhibits folate-dependent enzymes in the de novo synthesis of purines and pyrimidines [15, 16]. MTX is used as monotherapy for patients with early RA [17] and as an anchor drug for combination therapy with other DMARDs or biologics in patients with established RA who are MTX-insufficient responders [18]. However, long-term use of MTX leads to drug resistance and causes severe side effects such as nausea, neutropenia, pulmonary fibrosis, and hepatitis [15, 19].

Nanoparticles (NPs) are promising therapeutics due to their ability to deliver and release drugs [20, 21]. Multiple NPs have been developed for drug delivery in various diseases. They can inhibit fast excretion of drug from body by sustained release. NPs are typically administered by intravenous injection, oral feeding, or subcutaneous injection. Intravenously injected NPs are rapidly effective but also rapidly excreted and have a high risk of side effects. Oral feeding is the easiest and low risk, but the absorption rate is too low. Subcutaneous injection shows moderate effectiveness in relation to these methods, so has an advantage in occupying the middle ground. However, most NP studies for RA therapy have used intravenous injection and oral feeding.

MTX-loaded nanoparticles (MTX-NPs) ameliorated murine model of experimental arthritis after subcutaneous injection. They were formulated with hydrophobic poly (D, L lactide-co-glycolide) (PLGA) and amphiphilic polyvinyl alcohol (PVA). MTX was stably loaded into the NPs. MTX-NPs attenuated the severity of murine model of experimental arthritis and reciprocally regulated Th17 and regulatory T and B cells in vivo.

## Methods

### Materials

Resomer RG 502 H PLGA and PVA (MW 30,000–70,000) were purchased from Sigma Aldrich (St. Louis, MO, USA). Dimethyl sulfoxide, 99.0% (methyl sulfoxide, DMSO) was purchased from Samchun (Seoul, Gangnam-gu, Korea).

### Preparation and characterization of MTX loaded nanoparticles

PLGA (50 mg) and methotrexate (5 mg) were dissolved in DMSO at 60 ℃. The solution was added dropwise to an aqueous solution of 1% PVA (w/v). The solution was homogenized at 7000 RPM for 2 min using a homogenizer (Ultra Turrax® T-25 homogenizer; IKA®-Werke, Staufen, Germany). After homogenization, non-encapsulated substances were removed by dialysis in distilled water for 1 h through a 14,000 molecular weight cut-off membrane. The size distribution of MTX-NP was measured in PBS using a Zetasizer Nano ZS90 (Malvern Instruments, Malvern, UK) at 25℃. Encapsulated MTX was quantified by assaying the absorbance of MTX at 370 nm and encapsulation efficiency was calculated by the formula: ((amount of encapsulated drug/amount of added drug) × 100%). MTX-NP was placed in a dialysis bag and immersed in a container containing 50 mL of PBS to analyze the drug release profile. At predetermined time points, 200 μL of external PBS were removed and the absorbance at 370 nm of MTX was measured using Microplate Reader Synergy H1 (Bio-Tek, USA).

### Toxicological analysis

Mice were injected subcutaneously with vehicle, free MTX (2.5 mg/kg) or MTX-loaded nanoparticle (2.5 mg/kg based on MTX). Twenty-four hours post-injection, blood samples were collected from the heart of mice under respiratory anesthesia. The blood sample for complete blood count (CBC) was placed in EDTA tube and CBC was measured using HEMAVET 950FS blood cell counter (Drew Scientific, Inc., Dallas, TX, USA). The blood samples for blood chemistry analysis were placed in heparin tubes and plasma were isolated. Creatinine aspartate aminotransferase (AST) and alanine aminotransferase (ALT) in plasma were measured by Fuji Dri-Chem NX500 (Fujifilm Corporation, Tokyo, Japan).

### CIA induction and treatment with nanoparticles

Six-week-old male DBA/1 J mice were purchased from Orient Bio Inc. (Seongnam, Korea). CIA was induced as described previously [22]. To induce CIA in mice, CII was dissolved overnight in 0.1 N acetic acid (4 mg/mL) with gentle rotation at 4 °C. DBA/1 J mice were injected intradermally at the base of the tail with 100 μg of CII emulsified in Freund’s adjuvant (Chondrex). Two weeks later, 100 μg of type II collagen dissolved and emulsified 1:1 with incomplete Freund’s adjuvant (Difco) was administered to the hind leg of mice as a booster injection. On day 24 after the first immunization, mice were injected subcutaneously with 2.5 mg/kg MTX or MTX-NPs twice weekly. Animals were maintained under specific pathogen-free conditions at the Institute of Medical Science of the Catholic University of Korea and were fed standard mouse chow and water. All experimental procedures were examined and approved by the Animal Research Ethics Committee of the Catholic University of Korea; the procedure conformed to all National Institutes of Health of the United States guidelines (Permit number: 2020-0067-01).

### Clinical assessment of arthritis

To assess the severity of joint inflammation, the arthritis index was scored twice weekly from the onset of arthritis for up to 8–10 weeks after the primary immunization. The severity of arthritis was assessed on a scale of 0–4 according to the following criteria, as described previously [23]: 0 = no edema or swelling, 1 = slight edema and erythema limited to the foot or ankle, 2 = slight edema and erythema from the ankle to the tarsal bone, 3 = moderate edema and erythema from the ankle to the tarsal bone, and 4 = edema and erythema from the ankle to the entire leg. The arthritic score for each mouse was expressed as the sum of the scores of four limbs. Incidence was calculated as 25 percent of the presence of arthritis symptoms in one foot.

### Antibodies

The following antibodies were used for immunohistochemistry: rabbit polyclonal anti-TNF-α (#ab6671) and rabbit polyclonal anti-vascular endothelial growth factor (VEGF) (EP1176Y, #ab52917) antibodies were from abcam; rabbit polyclonal anti-IL-1β (#NB600-633) antibody was from Novus Biologicals. The following antibodies were used for flow cytometry: rat monoclonal APC anti-CD45R (B220) (RA3-6B2, #17-0452-83), mouse monoclonal PE anti-CD95 (APO-1/Fas) (15A7, #12-0951-81), mouse monoclonal PerCP-Cyanine5.5 anti-CD19 (SJ25C1, #45-0198-42), rat monoclonal FITC anti-CD5 (53-7.3, #11-0051-82), rat monoclonal PE anti-CD1d (1B1, #12-0011-82), rat monoclonal APC anti-IL-10 (JES5-16E3, #17-7101-82) antibodies were from Thermo Fisher Scientific; rat monoclonal PE-Cy™7 anti-CD19 (ID3, #552854) antibody was from BD Biosciences; Alexa Fluor® 488 anti-GL7 (GL7, # 144612) antibodies was from BioLegend. The following antibodies were used for confocal microscopy: rat monoclonal Alexa Fluor® 488 anti-CD4 (GK1.5, #100423) and rat monoclonal APC anti-CD25 (PC6, #102012) antibodies were from BioLegend; rat monoclonal PE anti-IL-17A (eBio17B7, #12-7177-81) antibody was from Thermo Fisher Scientific; mouse monoclonal PE anti-STAT3 (pY705) (4/P-STAT3, #612569) antibody was from BD Bioscience; rabbit polyclonal PE anti-Foxp3 antibody (#NP100-39002) antibody was from Novus Biologicals.

### Histology

Mouse joint tissues were fixed in 10% neutral-buffered formalin, decalcified in a decalcifying agent (National Diagnostics, Atlanta, GA, USA), embedded in paraffin, and sectioned. The sections (5 μm thick) were stained with hematoxylin and eosin (H&E) and scored as described previously [24]. Inflammation was scored using the following criteria: 0, no inflammation; 1, slight thickening of the lining or infiltration of some cells into the underlying layer; 2, slight thickening of the lining with infiltration of some cells into the underlying layer; 3, thickening of the lining, with an influx of cells into the underlying layer and cells evident in the synovial space; and 4, extensive infiltration of the synovium by inflammatory cells. Cartilage damage was evaluated by staining with Safranin O and toluidine blue, and the extent of damage was scored using the following criteria: 0, no destruction; 1, minimal erosion (limited to single spots); 2, slight-to-moderate erosion in a limited area; 3, more extensive erosion; and 4, general destruction.

### Immunohistochemistry

Sections were treated with 3% (v/v) H_2_O_2_ in methanol to block endogenous peroxidase activity. Immunohistochemistry was performed using the Envision Detection™ kit (DAKO Agilent Technologies Inc., Santa Clara, CA, USA). Tissue sections were incubated with primary antibodies against IL-1β, TNF-α, and VEGF for 2 h at room temperature. Sections were then incubated with a biotinylated secondary antibody and streptavidin–peroxidase complex for 30 min. The final colored products were developed using chromogen diaminobenzidine. The sections were examined by light microscopy (Olympus, Tokyo, Japan). The number of positive cells was counted at high-power field (magnifications × 400) with the aid of Adobe Photoshop software by four individuals and averaged three randomly selected fields per tissue section.

### Isolation of splenocytes

Mouse spleens were ground using sterilized glass slides with frosted ends and red blood cells were lysed in hypotonic ACK buffer (0.15 mM NH_4_Cl, 1 mM KCO_3_, and 0.1 mM EDTA, pH 7.4). The remaining splenocytes were filtered through a 40 µm cell strainer (Falcon, Durham, NC) and maintained in RPMI 1640 medium containing 5% fetal bovine serum (ThermoFisher Scientific, MA, USA).

### Flow cytometry

For surface marker staining, single-cell suspensions were washed with FACS buffer and incubated with fluorochrome labeled-antibodies for 30 min at 4 °C. For intracellular staining, single-cell suspensions were cultured with 25 ng/ml phorbol 12-myristate 13-acetate (Sigma-Aldrich, St. Louis, MO, USA) and 250 ng/ml ionomycin (Sigma-Aldrich) with the addition of GolgiStop (BD Biosciences, Franklin Lakes, NJ, USA) for 4 h. After surface staining, cells were fixed and permeabilized with Cytofix/Cytoperm according to the manufacturer’s instructions (BD Biosciences). After washing with Perm/Wash buffer, antibodies for intracellular staining were added for 30 min at 4 °C. To determine the frequency of germinal center (GC) B cells, splenocytes were immunostained with Alexa Fluor® 488-conjugated anti-GL7 PE-conjugated anti-Fas, APC-conjugated anti-B220, and PerCP-Cyanine5.5 anti-CD19 antibodies. For regulatory B cells, splenocytes were immunostained with PE-Cy™7 anti-CD19, rat monoclonal FITC anti-CD5, PE anti-CD1d, and APC anti-IL-10 antibodies. Events were collected using the FACS Calibur (BD Biosciences) or CytoFLEX (Beckman Coulter), and the data were analyzed using Flow Jo software, v. 7.6 (Treestar, Ashland, OR, USA).

### Confocal microscopy

Spleen tissues were snap-frozen in liquid nitrogen and stored at − 70 °C. Tissue Sects. (5 μm thick) were fixed in acetone. To stain IL-17 + or phosphorylated (p)-STAT3 + in CD4 + cells, Alexa Fluor® 488-labeled anti-CD4, PE-labeled anti-IL-17A, and PE-labeled anti-p-STAT3 (pTyr705) antibodies were used. To stain regulatory T (Treg) cells, Alexa Fluor® 488-labeled anti-CD4, PE-labeled anti-Foxp3, and APC-labeled anti-CD25 antibodies were used. Sections were analyzed using the LSM 510 Meta Confocal Microscopy System (Carl Zeiss, Oberkochen, Germany). Positive cells were counted visually at high magnification by four investigators.

### Real-time polymerase chain reaction

Total RNA was extracted using TRI Reagent (Molecular Research Center, Inc., Cincinnati, OH, USA), and cDNA was synthesized with the Dyne First-Strand cDNA Synthesis Kit (Dyne Bio, Seongnam, Korea) according to the manufacturer’s protocol. Gene expression was measured using the StepOnePlus Real-Time PCR System (Applied Biosystems, Foster City, USA) with SYBR premix (Bioline USA Inc. Taunton, MA). The following primers were used: IL-1β, 5′-GGATGAGGACATGAGCACATTC-3′ (sense) and 5′-GGAAGACAGGCTTGTGCTCTGA-3′ (antisense); IL-6, 5′-AACGATGATGCACTTGCAGAAA-3′ (sense) and 5′-TCTGAAGGACTCTGGCTTTGTC-3′ (antisense); IL-17A, 5′-TTTAACTCCCTTGGCGCAAAA-3′ (sense) and 5′-CTTTCCCTCCGCATTGACAC-3′ (antisense); and β-actin, 5′-GTACGACCAGAGGCATACAGG-3′ (sense) and 5′-GATGACGATATCGCTGCGCTG-3′ (antisense). mRNA levels were normalized to that of β-actin mRNA.

### Statistical analysis

All statistical analyses were performed using Prism (v. 8 for Windows; GraphPad Software). P-values were calculated by two-tailed paired *t*-test and two-way analysis of variance (grouped). P < 0.05 was considered indicative of statistical significance.

## Results

### Preparation and characterization of MTX-NPs

**Fig. 1:**
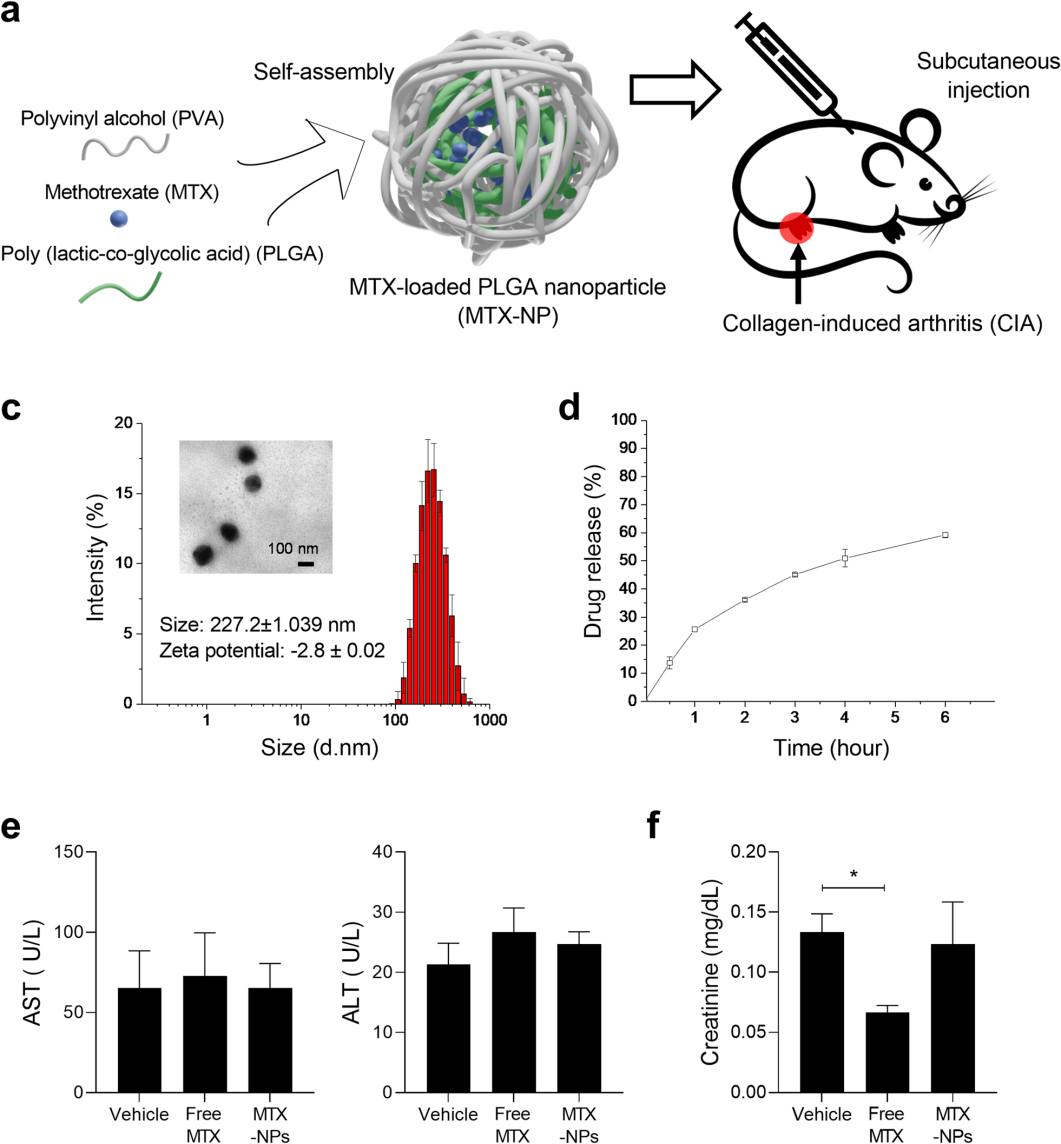
Characteristics of MTX-NPs. **a** Schematic of MTX-NP synthesis. **b** Size distribution, zeta-potential value, and TEM image of MTX-loaded nanoparticles. **c** MTX release profile of MTX-NPs. **d** Toxicological analysis of MTX-NPs in plasma. Plasma level of hepatotoxicity biomarker, aspartate aminotransferase (AST) and alanine aminotransferase (ALT). **e** Creatinine concentration, biomarker for kidney function. Error bar represents mean ± S.D. (n = 3). **P < 0.01 *vs*. control group

We prepared PLGA NPs via a homogenization method using PVA as stabilizer. MTX was physically loaded into the NPs (Fig. 1a). MTX was stably encapsulated in a hydrophobic core (PLGA) with an encapsulation efficiency of 76.6 ± 4.9%. The size of MTX-NPs was 227.2 ± 1 nm and MTX-NPs were spherical by transmission electron microscopy (TEM) (Fig. 1b). The size is suitable for injection into body by syringes with small needle. The polydispersity index (PdI) and zeta potential of MTX-NP were 0.139 ± 0.041 and − 2.8 ± 0.02, respectively, showing a homogeneous formulation without aggregation and near-neutral surface, respectively. In PBS at pH 7.4, free MTX was slowly released from the PLGA core (Fig. 1c), indicating sustained release (Fig 1d). To investigate side effect of MTX-loaded nanoparticle, we measured complete blood count (CBC), creatinine, aspartate aminotransferase (AST) and alanine aminotransferase (ALT). In CBC and liver function (AST, ALT), free MTX, MTX loaded NPs did not show any toxicity (Fig. 1e), (Table 1). Free MTX exhibited renal toxicity with low creatinine concentration, a typical side effect. However, in case of MTX-loaded NPs, the value was similar with that of control group, which means that NPs are useful for reducing renal toxicity of MTX (Fig. 1f).

**Table 1:** Complete blood count of mice which were subcutaneously injected with vehicle, free MTX or MTX NPs

	Normal range	Vehicle	Free MTX	MTX NPs
WBC (K/μL)	1.8–10.7	4.38 ± 1.81	5.27 ± 0.39	4.65 ± 1.59
NE (K/μL)	0.1–2.4	0.47 ± 0.23	0.79 ± 0.07	0.62 ± 0.06
LY (K/μL)	0.9–9.3	3.72 ± 1.47	4.29 ± 0.37	3.79 ± 1.4
MO (K/μL)	0.0–0.4	0.16 ± 0.09	0.16 ± 0.02	0.22 ± 0.14
EO (K/μL)	0.0–0.2	0.023 ± 0.015	0.017 ± 0.006	0.017 ± 0.015
BA (K/μL)	0.0–0.2	0.003 ± 0.006	0 ± 0	0.003 ± 0.006
NE (%)	6.6–38.9	10.51 ± 0.82	15.12 ± 0.73	14.03 ± 3.49
LY (%)	55.8–91.6	85.37 ± 1.71	81.39 ± 1.09	81.01 ± 3.37
MO (%)	0.0–7.5	3.55 ± 0.7	3.14 ± 0.73	4.55 ± 1.67
EO (%)	0.0–3.9	0.48 ± 0.23	0.3 ± 0.07	0.34 ± 0.21
BA (%)	0.0–2.0	0.08 ± 0.02	0.63 ± 0.01	0.077 ± 0.09
RBC (M/μL)	6.36–9.42	7.47 ± 0.57	7.67 ± 0.7	7.21 ± 0.33
Hb (g/dL)	11.0–15.1	12.17 ± 0.64	12.2 ± 0.89	12.17 ± 0.65
PLT (K/μL)	592–2972	776 ± 307	757.3 ± 223	792.7 ± 87.51
MPV (fL)	5.0–20.0	3.73 ± 0.06	3.77 ± 0.06	3.8 ± 0.17

*WBC* white blood cell (K/μL, 10^3^ cells/μL), *NE* Neutrophil, *LY* lymphocyte, *MO* monocyte, *EO* eosinophil, *BA* basophil, *RBC* red blood cell (M/μL, 10^6^ cells/μL), *Hb* hemoglobin, *PLT* platelet, *MPV* mean platelet volume (mean ± S.D., n = 3)

### MTX-NPs attenuate the severity of autoimmune arthritis

**Fig. 2:**
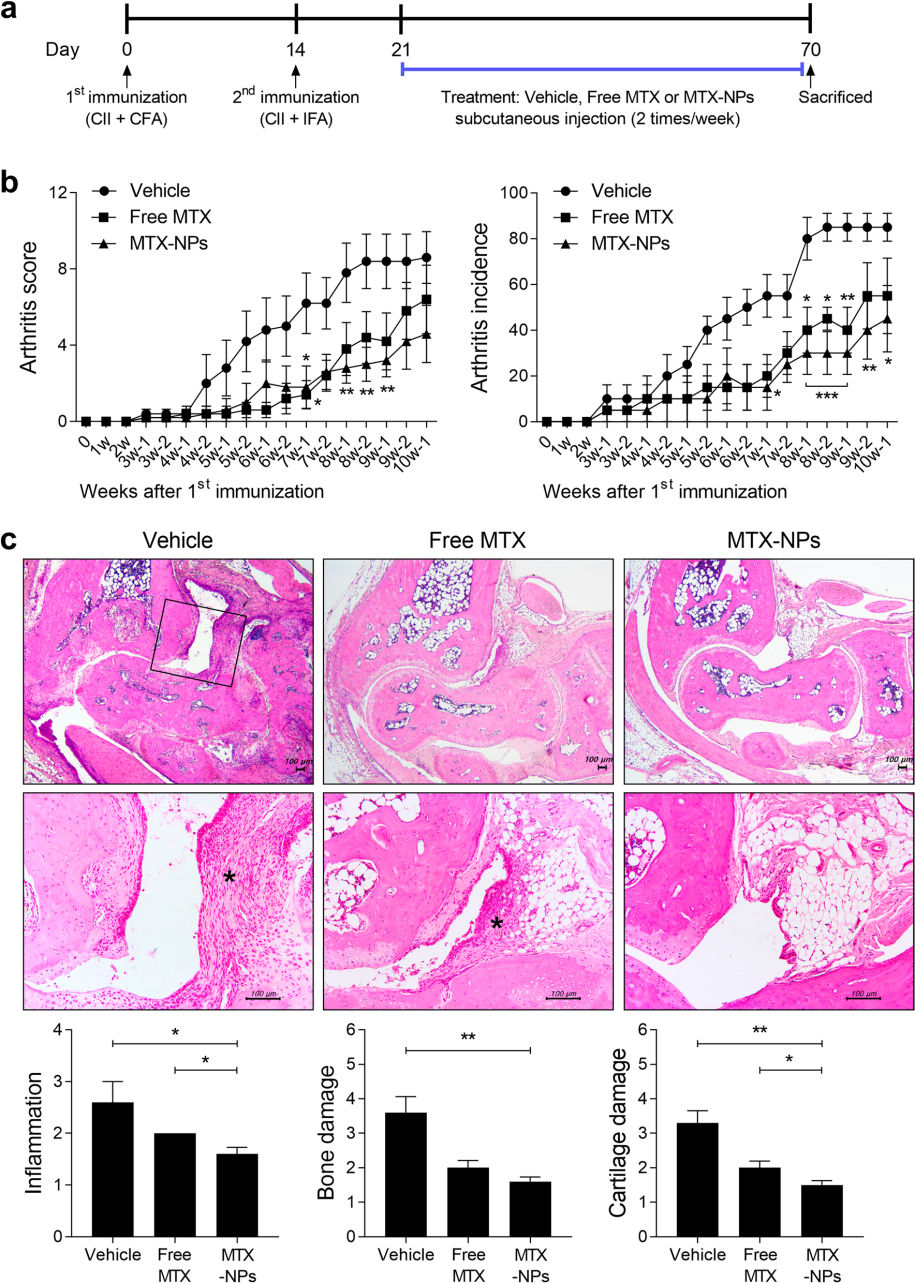
MTX-NPs ameliorated the severity of collagen-induced arthritis. **a** A graphic scheme of CIA induction and vehicle, free MTX or MTX-NPs administration. Beginning 3 weeks after the first immunization with type II collagen (CII), mice were injected subcutaneously with vehicle, free MTX, or MTX-NPs twice per week for 7 weeks (n = 5/group). **b** Arthritis score and incidence are shown for each group. **c** At 70 days after the first CII immunization, tissue sections from the paw and ankle joints of mice were stained with hematoxylin and eosin (original magnification × 40). Lower panels show enlarged view of the region within a box in the upper panels in each group. Asterisk: inflammatory cell infiltration. Representative histological features are shown. Graphs present quantified levels of inflammation, bone damage, and cartilage damage. Values are means ± SEM. *, P < 0.05, **, P < 0.01, and ***, P < 0.001 *vs*. control group. Data are representative of two independent experiments

**Fig. 3:**
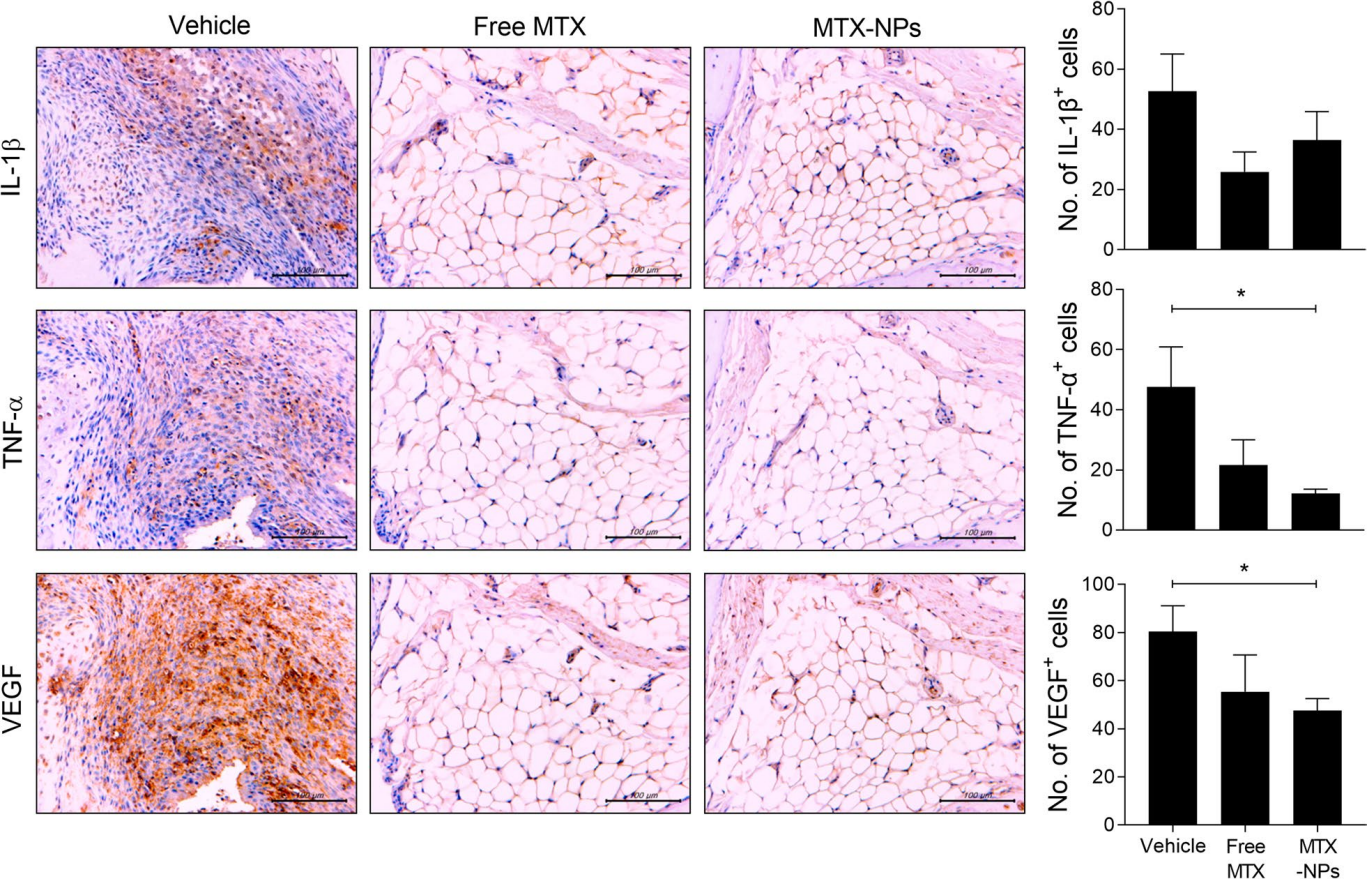
MTX-NPs suppressed the levels of inflammatory mediators in vivo. Beginning 3 weeks after the first immunization with type II collagen (CII), mice were injected subcutaneously with vehicle, free MTX, or MTX-NPs twice per week for 7 weeks (n = 5/group). At 70 days after the first immunization with CII, sections of joint tissues (*n* = 5/group) were stained with antibodies against interleukin (IL)-1β, tumor necrosis factor (TNF)-α, and VEGF. Graphs present numbers of antibody-positive cells for each cytokine. Data are means ± SEM of two independent experiments. *P < 0.05 vs. control group

To determine whether MTX-NPs could modulate the development of experimental model of arthritis in vivo, free MTX or MTX-NPs were administered to mice with CIA at 3 weeks after CII immunization (Fig. 2a). Subcutaneous injection of MTX-NPs in arthritic mice significantly reduced the arthritis score and incidence compared with vehicle-treated CIA mice. Injection of free MTX also reduced the arthritis score and incidence in CIA mice, but statistical significance was not consistently achieved (Fig. 2b). Histologic examination of joints stained with H&E showed that the ankles of MTX-NPs-treated mice exhibited less severe inflammation, bone damage, and cartilage damage compared with vehicle-treated mice. Application of MTX-NPs, in particular, exerted a more profound inhibitory effect on joint destruction compared with free MTX (Fig. 2c). Furthermore, the levels of inflammatory mediators—including IL-1β, TNF-α, and VEGF—were significantly lower in the joint sections from MTX-NPs-treated mice compared with vehicle-treated mice (Fig. 3).

### MTX-NPs reciprocally regulate the Th17 cells and Treg cells in vivo

**Fig. 4:**
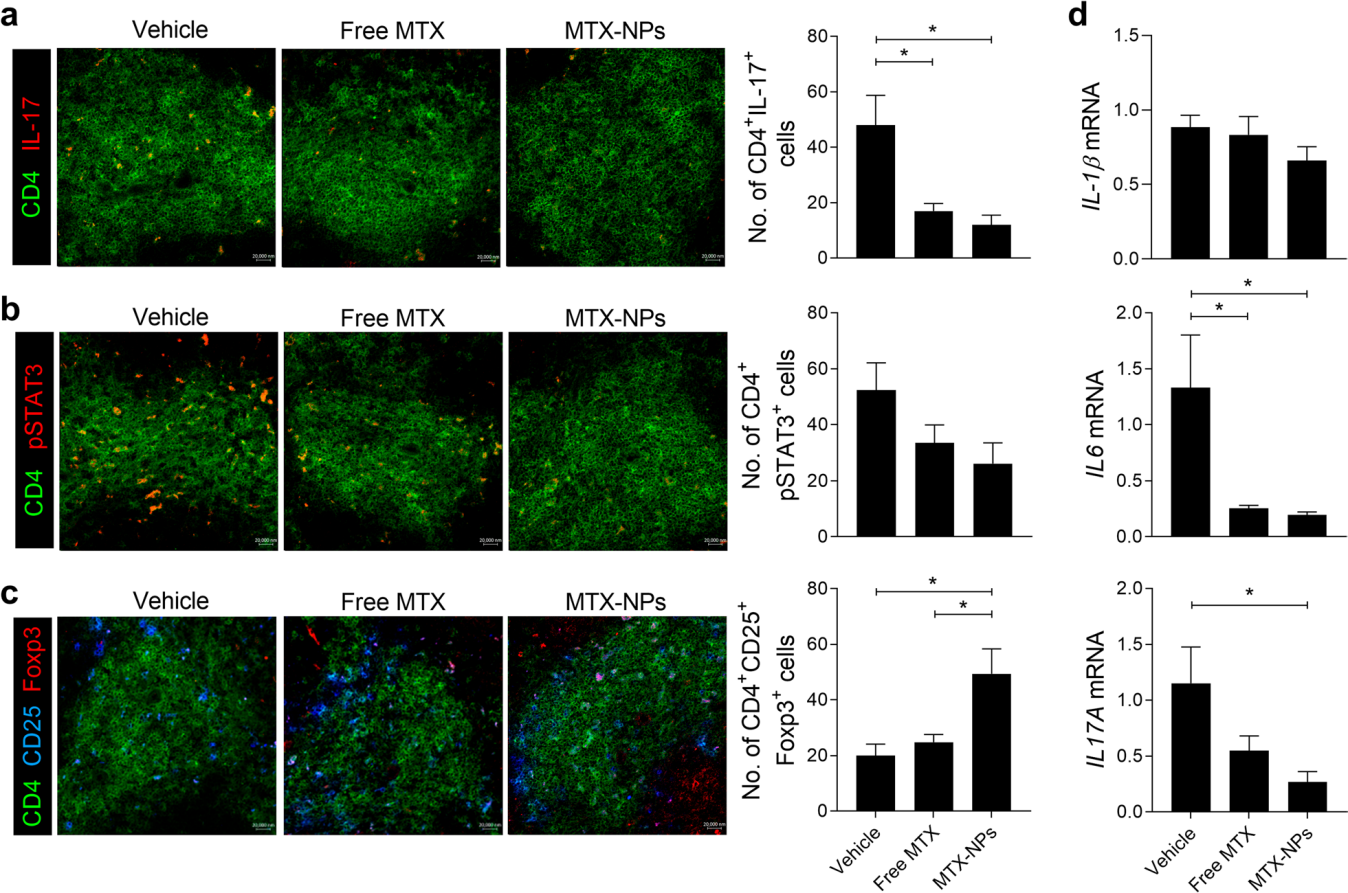
MTX-NPs regulate the number of Th17/Treg cells and expression of inflammatory cytokines in vivo. Beginning 3 weeks after the first immunization with type II collagen (CII), mice were injected subcutaneously with vehicle, free MTX or MTX-NPs twice per week for 7 weeks (n = 5/group). **a**–**c** At 70 days after the first immunization, spleen tissues were isolated and stained for CD4 + IL-17 + (**a**), CD4 + p-STAT3 (Y705) + (**b**), and CD4 + CD25 + Foxp3 + (**c**) cells. Cell subsets were analyzed in four independent quadrants by confocal laser microscopy. The distributions of the cell populations are shown. **d** At 70 days after the first immunization, ex vivo splenocytes were isolated and the mRNA levels of IL-6, IL-1β, and IL-17 were determined by real-time PCR. Data are means ± SEM of two independent experiments. *P < 0.05 *vs*. control group

To evaluate whether MTX-NPs suppress Th17 cells in vivo, the number of CD4 + IL-17 + Th17 cells in the spleens from CIA mice injected with MTX-NPs was investigated by confocal microscopy. The number of Th17 cells was lower in MTX-NPs- or free MTX-treated CIA mice compared with vehicle-treated CIA mice (Fig. 4a). STAT3 phosphorylation in CD4 + cells decreased in MTX-NPs- or free MTX-treated CIA mice compared to vehicle-treated CIA mice, but there was no statistical significance (Fig. 4b). To investigate whether MTX-NPs reciprocally regulate the population of Th17 and Treg cells in vivo, the number of CD4 + CD25 + Foxp3 + Treg cells in the spleen was investigated. The number of Treg cells in the spleen of MTX-NPs-injected mice significantly increased compared to vehicle-injected mice, and the increase was also significant compared with free MTX-injected mice (Fig. 4c). In addition, the mRNA levels of IL-6 and IL-17A, inflammatory cytokines related to Th17 cell development, significantly decreased in ex vivo splenocytes of mice injected with MTX-NPs compared to mice injected with vehicle (Fig. 4d).

### MTX-NPs increase regulatory B cells in vivo

**Fig. 5:**
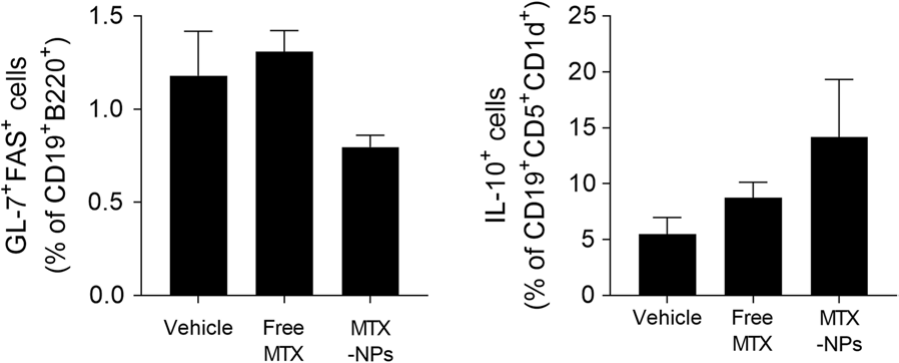
MTX-NPs increased the number of regulatory B cells in vivo. Beginning 3 weeks after the first immunization with type II collagen (CII), mice were injected subcutaneously with vehicle, free MTX, or MTX-NPs twice per week for 7 weeks (n = 3/group). At 70 days after the first immunization, ex vivo splenocytes were isolated and the number of CD19 + B220 + GL-7 + FAS + and CD19 + CD25 + Foxp3 + cells determined by flow cytometry. Values are percentages of positive cells

To evaluate whether MTX-NPs act on B-cell responses in vivo, we examined the number of ex vivo CD19 + B220 + GL-7 + Fas + GC B cells and CD19 + CD5 + CD1d + IL-10 + regulatory B (Breg) cells in splenocytes from CIA mice injected with MTX-NPs by flow cytometry. The number of GC B cells was lower whereas the number of regulatory B cells was higher in ex vivo splenocytes from MTX-NPs-injected CIA mice compared with vehicle-injected mice, but there was no statistical significance (Fig. 5).

## Discussion

We investigated the therapeutic potential of MTX-NPs administered subcutaneously in a murine model of autoimmune arthritis. MTX-NPs significantly reduced the clinical and histologic severity of experimental model of arthritis. MTX-NPs reduced the number of TNF-α– and VEGF–positive cells and reciprocally regulated the number of Th17 and Treg cells in the spleen from MTX-NP-treated CIA mice. Moreover, injection of MTX-NPs showed a tendency to increase the number of regulatory B cells in ex vivo splenocytes of CIA mice.

Synovial inflammation in RA involves infiltration of inflammatory cells, including monocytes/macrophages, B cells, and T cells, which are sources of chemokines and inflammatory cytokines [25]. T cells are a key player in the inflamed joints of patients with RA [26]. Although most subsets of CD4 + T cells are involved in RA pathogenesis, Th17 cells in particular positively correlate with disease activity in RA [27]. In CIA mice, deficiency of IL-17 suppressed the development of arthritis and reduced the production of type II collagen-specific immunoglobulin [28]. Moreover, blockade of IL-17 ameliorated the severity of arthritis and prevented synovial inflammation and joint destruction in CIA mice [29]. MTX exerts a potent therapeutic effect by modulating humoral and cellular immune responses in RA management [14, 30]. MTX increases the sensitivity of T cells to apoptosis [16] and inhibits NF-κB activation in T cells via tetrahydrobiopterin 4 depletion and JNK activation [31]. MTX suppresses the expression of IL-6 and IL-6–driven proliferation of fibroblast-like synoviocytes from patients with RA [32]. Thomas et al. demonstrated that MTX suppressed the JAK/STAT pathway in the Drosophila system and human macrophage lines [33], but the effect of MTX on JAK/STAT signaling and Th17 and Treg frequencies in the CIA model are unclear. The number of Th17 cells in the spleen of CIA mice decreased by injection of free MTX, and the effect was increased by injection of MTX-NPs.

The importance of B cells in RA pathogenesis has been demonstrated. B-cell-deficient mice had impaired CIA development [34], and B-cell depletion by an anti-CD20 antibody delayed CIA development [35]. Immunization with type II collagen induced the formation of germinal centers in lymph nodes, which was necessary for CIA development [36]. Breg cells control autoimmune diseases by secreting anti-inflammatory cytokines (*e*.*g*., IL-10 and IL-35) and transforming growth factor-β [37, 38]. Moreover, Breg cells suppress the differentiation of proinflammatory lymphocytes (e.g., Th1 and Th17 cells) and dendritic cells [39]. The frequency of Th17 cells and germinal center B cells decreased in ex vivo splenocytes of MTX-NP-treated CIA mice.

The elimination half-life of free MTX at the injection site in human is 0.16 h in case of subcutaneous injection [40]. Our NPs provided sustained release of MTX and increased the residence time. Although the release of MTX from NPs was faster than in prior reports, it affected the therapeutic results significantly. It is necessary to test and optimize further the release pattern of MTX by changing NP size or composition. In addition, MTX in NP could be taken up by T cells and B cells before release. This is because the NP surface was coated with PVA; we did not use polyethylene glycol groups to prevent fouling and unwanted uptake into immune cells. In addition, further modification with biological ligands will provide information for specific targeting of NPs to particular cell types.

## Conclusion

In summary, our study showed a therapeutic efficacy of MTX-NPs in mice with CIA. Subcutaneous injection of MTX-NPs effectively alleviated the development of experimental model of arthritis and suppressed the infiltration of inflammatory factors-expressing cells in the joint from CIA mice. Moreover, the number of pathogenic Th17 cells and inflammatory factors including TNF-α decreased while the number of regulatory T cells increased in the spleen of the MTX-NPs-injected group. These findings suggest that MTX-NPs have potential as a more advanced therapeutic strategy to overcome the limitations of MTX therapy.

## Data Availability

All data are available in the manuscript or upon request to the authors.

## Abbreviations

RA: Rheumatoid arthritis
TNF: Tumor necrosis factor
IL: Interleukin
Th17: IL-17-producing T helper
MTX: Methotrexate
DMARD: Disease-modifying antirheumatic drug
NP: Nanoparticle
PVA: Polyvinyl alcohol
PLGA: Poly (d, l lactide-co-glycolide)
H&E: Hematoxylin and eosin
VEGF: Vascular endothelial growth factor
GC: Germinal center
P: Phosphorylated
Treg: Regulatory T
TEM: Transmission electron microscopy
PdI: Polydispersity index
Breg: Regulatory B
